# Perspectives of the Young Cardiovascular Surgeon

**DOI:** 10.21470/1678-9741-2020-0350

**Published:** 2020

**Authors:** Luís Alberto O. Dallan, Fabio B. Jatene

**Affiliations:** 1 Cardiovascular Surgery Division, Instituto do Coração do Hospital das Clínicas da Faculdade de Medicina da Universidade de São Paulo (InCor-HC-FMUSP), São Paulo, SP, Brazil.

**Based on the closing speech given by Prof. Luis Dallan, honored by the class of fourth-year residents in March 2020.**

**This message is addressed to young doctors who complete their Cardiovascular Surgery Residency and start their surgical lives independently, often already working in their own service.**

## Medicine and the Evolution of the Times

One of the most widely accepted theories suggests that our universe originated approximately 13.7 billion years ago^[1]^. The current degree of evolution of our species, in turn, would have occurred in the last 300 million years^[2]^.

Despite this long evolutionary period, until the middle of the 19^th^ century, science was empirical and inefficient, as well as the work of doctors. Hospitals did not exist, or were mere places where charity was practiced, and moral comfort was provided for suffering. Pharmacology based on elixirs, teas and herbs did not survive the entry into the 20^th^ century, as only two medicines from the 19^th^ century reached the next century: methylene blue and aspirin.

The health professional was represented by the family physician, who, despite its limitations, had all respect, consideration, and sometimes even veneration. However, his position before society started to change from the second half of the 19^th^ century, with new events, such as vaccination. More precisely, in 1885 Louis Pasteur developed the anti-rabies vaccine, generating the first result of great repercussion for microbiology applied to medicine^[3]^.

But, undoubtedly, the great technoscientific advance took place after the Second World War. Despite the devastation and the need to reconstruct many locations in Europe, in the United States the end of World War II was accompanied by a great scientific evolution. Much of this technological development, especially in the area of nuclear weapons and computing, has been used in diagnostic research and medical therapy. Many other advances in different fields followed, such as genetics, molecular medicine, better understanding of diseases and, obviously, cardiology.

## Cardiac surgery is a separate case

Cardiac surgery had begun slowly in the 1940s, with some operations that did not require cardiopulmonary bypass, such as closure of a patent ductus arteriosus, coarctation repair and, at the end of the decade, mitral commissurotomy. In 1945, Dr. Alfred Blalock, with Dr. Helen Taussig, performed a cardiac surgery in a child with congenital heart disease at the Johns Hopkins Hospital in Baltimore^[4]^. In the early 1950s, a defect in the atrial septum was closed only with the use of hypothermia. It was clear, however, that there was a need for auxiliary devices that allowed direct access to the interior of the cardiac chambers.

In that same decade, Dr. John Gibbon extended his studies with artificial oxygenators and created what he called an “heart-lung machine”, establishing the principles of cardiopulmonary bypass^[5]^. This was the “start” for this new specialty that we now embrace, as it was followed by multiple actions on valves and, finally, on the coronary arteries.

We were privileged to have not only teachers, but also people with ideology and initiative that put us at the forefront of the specialty world. Just to name two, we remember Prof. Zerbini, to whom we owe the conception and materialization of the Heart Institute (InCor), which bears his name, and Prof. Jatene, who among many other contributions, offered the world with the surgical treatment of transposition of the great arteries^[6]^. Specifically, InCor was also directed by distinguished surgeons, such as Prof. Sérgio Almeida de Oliveira, Prof. Noedir Stolf and currently Prof. Fabio Jatene. We could mention countless others in Brazil and Latin America, but remember that this is the moment for their disciples, many of them from this house, like Dallan, Pablo and all the other assistants.

## Today’s Cardiac Surgery and the Medicine of the Future

The enthusiasm of the fantastic knowledge of a simple mechanical pump has given the heart and its surgical correction the status of the most studied scientific area worldwide and at all times. All of this is supported by the rear of more sophisticated diagnostic means, such as the development of hemodynamic studies and machines that allowed to see its functioning and its essence. The heart started to have an accurate diagnosis of its problems and adequate treatment, but without ever losing the “glamor” of being “the carrier of human emotions”.

On the other hand, these advances have led to unfavorable factors. Perhaps the biggest one is due to the new technology facilitating less involvement of doctors with patients, reducing their direct relationship with them. Add to that, the adverse economic conditions and the widespread attendance, situations that can contribute to undermine this adequate relationship, making this involvement more impersonal.

All these factors, however, did not prevent the growing development of cardiac surgery. Its evolution, however, was not linear. For example, initial procedures used in myocardial revascularization, such as the use of the mammary artery and the non-use of cardiopulmonary bypass, are still considered today in the surgical treatment of coronary disease.

In our view, it is not a question of a lack of options or innovations, but that the initial procedure was a very advanced technology and sophistication for the time. We do a parallel to aviation. Today we fly commercial planes with the same design and speed as those of the 1960’s. Only the seat distance has become narrower. Aviation has not evolved? We prefer to reason that the initial technology was spectacular and will take decades to be overcome!

More than 1,000,000 cardiac surgeries per year are performed worldwide. New methods also proposed have not displaced cardiac surgery. Coronary angioplasty, which emerged in the 1980s as a redemptive and a threat to surgical treatment, was established, but soon presented its limitations. Percutaneous procedures, such as treatment of aortic aneurysms and transcutaneous aortic valve replacement (TAVR), also occupy their space and are certainly being incorporated into the surgeon’s arsenal. Today we observe that, in practice, the “charm and mystique that cardiac surgery provides” are still present.

We cannot untie the future of cardiac surgery with that of medicine. How will both fit into the 21^st^ century? Certainly, the three pillars that support medical practice will be maintained, namely, prevention, diagnosis and objective therapy. The technical-scientific advance has no return and, possibly, will maintain its exponential growth, supported by the theoretical and scientific bases already established. The structure of evolution is likely to be based on genetics, immunology, nanotechnology, cybernetics and bioengineering.

In historic Sparta, human selection was achieved by eliminating the physically disabled. I believe that, in the future, this eugenics will happen through genetics, through the knowledge of sperm and egg codes, with adjustments by bioengineering. This will make it possible to avoid certain congenital diseases, resulting from chromosomal and metabolic disorders^[7]^. On the other hand, diseases with possible genetic transmission, such as diabetes, muscular dystrophies and certain types of cancers, can be prevented by genetic correction.

In diagnosis, the evolution must be fantastic. It will include the development of computers with artificial intelligence and neural behavior^[8]^. It is believed that, around 2040, the degree of intelligence of these computers will be compatible or superior to the human intelligence itself, which for us will not be surprising, after one of them beat Garry Kasparov, world chess champion.

In the future, the diagnosis should occur “from the inside out”, with an unimaginable forecast. This will be possible through the development of nanotechnology, building miniaturized computers, reduced to sizes smaller than human cells^[9]^. Once inserted into the bloodstream, these miniaturized robots can be located directly at the diseased point, providing ultraprecise data to the computer and observers, making it possible to reach a diagnosis, which would be unlikely through external methods.

The “icing on the cake” will be reserved for therapy! Its reformulation will involve pharmacology, with new genetic foundations and new molecular creations. Currently, drug production is carried out at an industrial level. This will allow for different clinical responses, arising from different individual genetic codes of people.

Possibly future treatments will be individualized, with more potent medications appropriate to the profile of each person. This may even involve genetic overtones of certain breeds. The traditional generic package will end, and each drug will be determined by computers, which will define its dosage, the speed of administration flow, carried through nanobots and directly to the disease site. In addition to enhancing therapy, this will reduce or eliminate side effects. Today, successful experimental models in mice, whose technology has made it possible to cure diabetes, are already being put into practice.

It will be up to the development of nanobots to supplement the functional deficiencies resulting from aging, correcting them, reinforcing them, or even replacing them with ideally predicted substances.

Raymond Kurzweil, when he wrote his book *The age of intelligent machines*, stated that, in the future, we will all be, to some degree, cyborgs!^[10]^ In a way, on a daily basis, we come across people with prostheses in the lenses, femoral prostheses, hips, aortic, hearing aids, coronary stents and we await new developments for the disabled.

Although practically untouched, the human brain is already the focus of some incursions, especially peripheral ones. The challenge will be to mimic its performance through its millimeter mapping. Therefore, supercomputers study, copy and seek to reproduce their functionality through microchipping. They would become true neural or neuronal computers, capable of performing all mental and psychic functions. In a first step, thousands of neuronal nanobots could be positioned in damaged brain regions, aiming to revitalize or replace them, to compensate for deficiencies or absences. This would not be reserved only for cases of cerebral ischemia but extended to cases of autism or other neuropathies. But diseases such as Parkinson’s, schizophrenia, Alzheimer’s and countless neuropsychic disorders will surely have a better approach.

It remains to be seen whether aging can be contained. The prospect is that the same therapy, with the implantation of thousands of nanobots in the brain, can be used. This would generate a hybrid organ of biological and non-biological intelligence, with artificial neurons replacing declining functions and activities.

## Cardiac Surgery of the Future

If the panorama described above materializes, life expectancy for the 22^nd^ century could rise to up to 140 years with, certainly, numerous problems resulting from such survival, whether social or health itself, and then cardiac surgery will gain greater emphasis.

But in the short term, how would cardiac surgery behave? Cardiac surgery will possibly be aided by robots, as is currently suggested. It can be carried out remotely, via satellite, with a command co-guided by intelligent computers^[11]^. Although this artificial intelligence can play a prominent role, the presence of the surgeon will remain essential, as a coordinating and preponderant figure in the surgical intervention. Despite the help in the scientific function offered by technology and as much support as the machine has, the medical art of treating patients, earning their trust and offering moral support, will never be overcome.

## Advice to the Young Surgeon

- The surgery must be planned with a Heart Team: surgeon, clinician and hemodynamicist. We must consider them allies in the indicated patients, especially if they are complex cases.
- Talk to the patient’s family, presenting the risks of the procedure. Those who come from a university hospital are usually not used to it.
- Integrate the anesthetist in the context of each surgery, as well as the perfusionist.
- Use CPB routinely, unless only the arteries of the anterior wall of the heart are treated. Do not attempt, at the beginning of your career, to perform all revascularizations without CPB.
- Monitor the patient in the immediate and late postoperative period. Do this routinely, which will certainly avoid numerous problems and improve results.
- Do not inhibit yourself from getting advice from those who have been through it all. A phone call often resolves the issue.

Good luck and success to all!!!

*In memoriam of Prof. Jayme Rozenbojm - a medical education enthusiast.*
